# The Emerging Potential of Multi-Ion Radiotherapy

**DOI:** 10.3389/fonc.2021.624786

**Published:** 2021-02-22

**Authors:** Daniel K. Ebner, Steven J. Frank, Taku Inaniwa, Shigeru Yamada, Toshiyuki Shirai

**Affiliations:** ^1^ National Institute of Radiological Science (NIRS), National Institutes of Quantum and Radiological Science and Technology (QST), Chiba, Japan; ^2^ Division of Radiation Oncology, The University of Texas MD Anderson Cancer Center, Houston, TX, United States

**Keywords:** heavy-ion radiotherapy, carbon-ion radiotherapy, helium-ion irradiation, radiation therapy, multi-ion radiotherapy

## Abstract

Research into high linear energy transfer (LET) radiotherapy now spans over half a century, beginning with helium and deuteron treatment in 1952 and today ranging from fast neutrons to carbon-ions. Owing to pioneering work initially in the United States and thereafter in Germany and Japan, increasing focus is on the carbon-ion beam: 12 centers are in operation, with five under construction and three in planning. While the carbon-ion beam has demonstrated unique and promising suitability in laboratory and clinical trials toward the hypofractionated treatment of hypoxic and/or radioresistant cancer, substantial developmental potential remains. Perhaps most notable is the ability to paint LET in a tumor, theoretically better focusing damage delivery within the most resistant areas. However, the technique may be limited in practice by the physical properties of the beams themselves. A heavy-ion synchrotron may provide irradiation with multiple heavy-ions: carbon, helium, and oxygen are prime candidates. Each ion varies in LET distribution, and so a methodology combining the use of multiple ions into a uniform LET distribution within a tumor may allow for even greater treatment potential in radioresistant cancer.

## Introduction

Seventy years have passed since Lawrence and Tobias first employed helium and deuteron particle beams in human patients, beginning the clinical study of charged particle radiotherapy (CPT), or hadrontherapy (1, 2). Their results built off the pioneering experience of Stone and colleagues, who treated 226 patients with neutrons from 1936 to 1938, with efforts only abridged by World War II (3, 4). As research resumed following the war, and a deeper understanding of radiobiology and the role of linear energy transfer (LET) developed, Catterall and colleagues began neutron radiotherapy treatment at Hammersmith Hospital in London in 1965.

While neutron radiotherapy exhibits high-LET, its conventional dose distribution limited the beam due to inherent toxicity unmitigated by fractionation (5). CPT was viewed as a viable alternative, combining high-LET with the Bragg peak, a physical characteristic of ion radiotherapy in which dose is deposited at an inverse of particle energy (6). This combination of physical and radiobiological advantages over conventional radiotherapy leads to an enhancement of the therapeutic ratio (7), with areas of higher LET experiencing higher relative biological effect (RBE); that is, an equal physical dose of CPT will have a resultant increased effect compared with conventional radiotherapy.

This expanded interest localized at the Lawrence Berkeley National Laboratory (LBNL). Following commissioning of the Bevalac in 1971, evaluation of fast neutron, proton, and negative pions, as well as helium, carbon, neon, nitrogen, silicon, and argon-ion beams began (3, 8). Each ion expresses high-LET regions within the particle travel path: the resultant distribution of LET varies between particles, as does the theorized physical dose distribution advantage. High-LET regions range from extreme distal in proton (9) to increasingly proximal with increase in atomic size (6). Each ion demonstrated benefits and limitations, with helium employed for improved dose localization and heavier-ions to amplify biological effect (10). Though initial work was promising, research ceased when the Bevalac and Bevatron were decommissioned in 1993 (8).

## Modern Particle Therapy

The advantages of particle radiotherapy are not without cost: the initial capital expense of particle centers is prohibitive and cost-benefit ratio remains a topic of considerable discussion (11–15). Moreover, in comparison with the relatively uniform ionization provided by conventional radiotherapy, with each ion comes varying considerations of intrabeam LET distributions, range uncertainty, lateral scattering, and distal fragmentation (16), as well as accurate dose deposition modeling along the beam path within varying tissues (17), and, finally, the combination of these factors into a treatment algorithm capable of providing a uniform biological effect within the treatment target. These latter elements, critically important for successful delivery of patient care, complicated the translation of photon doses into ion-beam treatment and informed the use of dose escalation clinical trials for determination of target and ceiling doses for histological sites (6).

Particle monotherapy has dominated discussion to date, and debate today continues: is there an ideal particle for treatment, and particularly for cost effective treatment? (18). When the Heavy Ion Medical Accelerator in Chiba (HIMAC) was constructed at the National Institute of Radiological Science (NIRS, Japan) in the early 1990s, prior experience at the Bevalac as well as previous usage of neutron at NIRS led to the selection of carbon-ions as the best ion for treatment, balancing considerations of particle size, center cost, and the perceived similarity of the RBE of the carbon-ion in its Bragg Peak to prior studies with neutron (6, 19).

Carbon-ion radiotherapy (CIRT) has remained under development for 30 years at the HIMAC, as well as at the German Society for Heavy Ion Research (Gesellschaft für Schwerionenforschung, GSI) and later Heidelberg Ion Beam Therapy Center (HIT), with significant physical, radiobiological, and clinical outcomes reported (6, 7). Clinical trials with CIRT have demonstrated enhanced kill effect in target tumors while simultaneously sparing normal tissue, with promising implications for reductions in secondary cancer incidence (20). The effect of the high-LET beam on traditionally radioresistant cancer has demonstrated unique promise, including in recurrent rectal cancer (21, 22), pancreas (23–25), glioma (26, 27), sarcoma (28–30), head-and-neck (31, 32), and others (7), with notable radiobiological effects seen in tissue *versus* conventional irradiation (33–35).

Nonetheless, CIRT can still result in imperfect local control, be this due to insufficient dose, uniquely radioresistant areas of the tumor [ranging from hypoxic regions (36) to isolated highly resistant stem-like cells (37)], or imperfect modeling of dose delivery (38). Other ions have demonstrated differing physical advantages, particularly with regard to variations in LET and physical dose distribution. After promising results at the LBNL, helium-ion radiotherapy has resurged through ongoing clinical translation at Heidelberg. Helium offers a decreased lateral scatter effect *versus* proton (39), with less fragmentation tail dose *versus* carbon. Kopp and colleagues have demonstrated single field setups with helium with an RBE_50%_ of 1.56 *versus* 2.16 for carbon in glioma, and 1.44 *vs.* 1.99 in ACC; this comes, however, with significantly lowered LET_50%_ (14.66 *vs.* 73.00 keV/micrometer in glioma and 13.39 *vs.* 64.92 in ACC). The authors concluded that combining the two would provide for more stable LET_d_ and RBE distributions *versus* monotherapy with either beam (40). Meanwhile, the United States has principally pursued proton irradiation with forays into LET optimization (NCT03750513), but has to date been unable to leverage the potential benefits of heavy-ion high-LET radiation in patient care.

## LET and RBE

The ability to sterilize a tumor is influenced both by physical dose delivered as well as the inherent LET of the beam path as it passes through the tumor. Heterogeneity of underlying tissue and the oxygen concentration within that tissue complicate the translation of target dose to cell-killing effect yet further, amplified by a current inability to study *in vivo* cell-level differences (41). Notably, the oxygen enhancement ratio, that is the particular radiation needed to result in equivalent treatment in the presence or absence of oxygen, is lower in particle therapy than with photon and generally increases with LET and decreases with atomic mass; this informs the apparent increased efficacy of heavy-ion radiotherapy in hypoxic conditions (19). To facilitate comparative utility between carbon-ion and photon irradiation, the RBE of a physical dose within a target was modeled: the Kanai model is experimentally derived and similar to the original models utilized at the Bevalac, defining RBE based on 10% survival of human salivary gland tumor cells under aerobic conditions (42). The microdosimetric kinetic model and local effect model were alternatively developed, the former for scanning CIRT in Japan and tuned to the Kanai model (43), while the latter was developed for European centers based on a biophysical modeling approach (44). Variations between the models have complicated regimen comparability between world centers (38).

Bassler and colleagues have demonstrated that even with equivalent dose distributions, substantially different LET distributions may be seen owing to LET dilution through secondary low-LET fragments generated through inflight nuclear reactions with increasing depth (45). The result is an equivalent dose delivered with variabilities in RBE. Clinically, this uncertainty has been effectively circumvented through dose escalation trials, tailoring dose delivered to levels of unacceptable toxicity. These phase I/II clinical trials have enabled the safe delivery of significantly ablative doses of CIRT (6), but with poor selectivity for delivering enhanced RBE to target regions within a tumor. LET/kill-painting is one method to bridge the gap between dose prescribed and cell kill-effect delivered: by optimizing and/or making uniform the high-LET region of CPT, oxygen effect may be minimized, thereby improving the uniformity of cell kill effect delivered (41). Moreover, low-LET regions of the beam could be preferentially relocated to healthy tissue, in theory further reducing healthy tissue toxicity (46). LET-weighted doses have effectively been demonstrated in proton radiotherapy (47).

The clinical relationship between LET distribution within a tumor and tumor control has been explored in CIRT. Hagiwara and colleagues studied the influence of dose-averaged LET on CIRT-irradiated pancreatic tumor control in 2020 (48), retrospectively evaluating 18 patients treated with 55.2 Gy (RBE) CIRT at a median of 22 months. Four infield central local recurrences were noted. While dose was uniform throughout the tumor, LET was lower within the central compartment of the target volume, owing to how particle paths were overlapped to generate a spread out Bragg peak. Notably, local control was improved in those patients with higher minimum dose-averaged LET within the gross tumor volume (GTV), independent of the minimal dose and D98 delivered (48). Improved dose-averaged LET within the GTV may thereby improve local control, though the ability to fully control the LET within the tumor target may be limited by the LET distribution inherent to the carbon-ion beam, and the tumor’s relationship to nearby radiosensitive organs.

Okonogi and colleagues similarly considered uterine cancer, focusing on whether LET was correlative with late rectal toxicity rate (49). In evaluating 132 patients with CIRT-treated uterine carcinoma and greater than 6 months of follow-up, nine were noted to have grade 3 or 4 late rectal complications. Regression analysis demonstrated an association with rectal D_2cc_, but not with dose-averaged LET nor physical dose. This echoes similar studies in proton that have demonstrated that LET and physical dose alone are poor correlative measures, and rather that the RBE-weighted dose is critical (50–52).

Seeking adequate base dose with strategically deployed high-LET irradiation led to initial combination studies, specifically modern CIRT-boost treatments (45) (p). Boost treatments typically combine CIRT or the lower-LET proton beam with intensity-modulated [photon] radiation therapy (IMRT), tomotherapy, or other forms of conventional radiotherapy (26, 53–59). Schulz-Ertner and colleagues in 2005 deployed carbon-boosted photon on adenoid cystic carcinoma to achieve three times the locoregional control of photon at 4 years (60). Another trial is investigating CIRT-boost for image-guided brachytherapy in locally advanced cervical cancer (61). Others have aimed to combine CIRT with proton, such as in one phase I/III trial on glioblastoma at the Shanghai Proton and Heavy Ion Center (62). Principally, the majority of modern high-LET clinical treatment has employed CIRT alone (6), and without LET optimization.

In seeking optimized LET within target tissues, Bassler and colleagues originated the concept of LET painting, building off the pioneering photon dose-painting concept by Ling et al. (63), and have evaluated multiple heavy-ions. For instance, LET-painting with carbon-ions allowed hypoxic subvolume control to a limit of 0.5 cm^3^; oxygen-ions, by comparison, could be extended to 2 cm^3^, and were further extendable with dose escalation (46). However, higher-LET oxygen-ion radiotherapy may cause increased normal tissue damage (41); no single ion has emerged as optimized for dose distribution, oxygen enhancement ratio (OER), nor overall translatability to hypoxic/radioresistant tumor kill effect.

Multi-ion radiotherapy (MIRT) theoretically provides for the benefits of each ion to be synergistically deployed in treatment. While intensity-modulated and LET-painted CIRT alone may achieve a high dose-optimized LET within a majority of tumor targets, the beam’s utility is limited by its inherent physical LET distribution. As such, incorporating the varying LET distributions of other heavy-ions into a dose-LET-optimized composite treatment plan may allow for new treatment options for patients with complex cancers. Lower-LET beams such as helium may offer improved margin dosage where tumors lie close to normoxic, healthy tissues, while higher-LET irradiation can be layered into hypoxic, radioresistant regions. Adaptive treatment planning would involve optimizing across multiple ions so as to achieve ideal cell-killing effect in the target tumor.

This is an easy vision to articulate, but decades in the making; innumerable challenges remain prior to trial development.

## Ongoing Development of MIRT

CIRT treatment at the NIRS-QST today combines pencil-beam raster (re)scanning (64–66), a phase-controlled 3D scanning irradiation system (67), motion management incorporating fast rescanning with respiratory gating (64, 68), and a superconducting gantry (69), enabling the conformal painting of heavy-ions voxel-by-voxel through a target and in theory the employment of combination dose- and LET-based treatment plans with LET/kill-painting of tumor tissue. Within this system, NIRS-QST plans to deploy helium, carbon, oxygen, and neon-ions for MIRT (70). Optimization of ion source insertion, and the rapid changing of sources, is critical for clinical throughput in a MIRT facility. This will use a single electron cyclotron resonance ion source (ECR-IS) with fast gas-switching operation. Particles are electron-stripped and then accelerated within a synchrotron, with beam purity assured due to mass separation owing to variation in mass-to-charge ratios. As helium-ions bear an equivalent ratio, Mizushima and colleagues developed a method to ensure beam purity prior to treatment using an ionization chamber and Faraday cup, with a contamination rate less than 1% (70).

**Figure 1:**
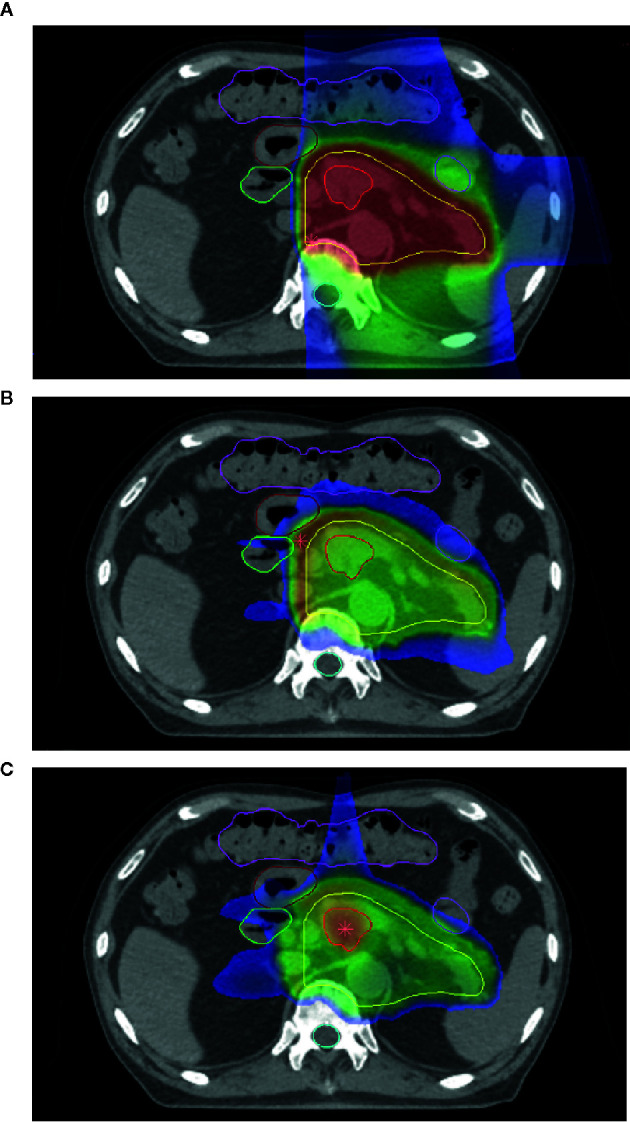
**(A)** Dose distribution of carbon-ion radiotherapy (CIRT) for a case of pancreatic cancer. **(B)** Linear energy transfer (LET) distribution of the same case. Note overlying LET distribution near organs-at-risk. **(C)** The LET of the same case combining helium, carbon, and oxygen-ion treatments using the intensity modulated composite particle therapy (IMPACT) system.

In 2016, Inaniwa and colleagues introduced a method to deliver two or more ion species in one treatment session, termed intensity modulated composite particle therapy (IMPACT) (71). By employing proton, helium, carbon, and oxygen ions, they were able to delineate valid prescribed LET ranges within a water phantom in opposing and orthogonal geometries. They further demonstrated the optimization method in a simulated prostate case, incorporating all four above ions. They were able to adjust the prostate, planning target volume, and rectum LETs to 80 keV/µm, 50 keV/µm, and below 30 keVµm, respectively, while maintaining dose in the PTV to a uniform 2 Gy. This served as proof of concept for the IMPACT system to dose and LET-average multiple ions within the treatment planning system at the NIRS. A demonstration of this system in pancreatic cancer may be seen in **Figure 1**, demonstrating the CIRT dose and LET distributions (**Figures 1A, B**), and the equivalent LET distribution using the IMPACT system (**Figure 1C**), with increased LET to 100 in the center field and decrease of OER from 2.8 to approximately 1.5. However, the IMPACT system at that time did not describe biological effect, and the authors noted that further development was required prior to beginning clinical trialing.

Inaniwa et al. additionally explored the nuclear interactions of particles within patients, adapting and validating the previously described planar integrated dose distribution measured in water (PID) correction method for scanned CIRT for treatment plans involving helium-, carbon-, oxygen-, and neon-ion beams (72). They similarly verified the stochastic microdosimetric kinetic model following previous work to optimize computational time and memory space, and verified the model within two cell irradiation experimental models, HSGc-C5 and MIA PaCa-2, which have notably different radiation sensitivities, for hypofractionated MIRT (73). This enables treatment planning of hypofractionated MIRT within treatment systems utilizing the MK model, principally centers in Japan.

At GSI, Scifoni et al. in 2013 developed an initial method for including OER in ion beam treatment planning (74, 75). Following the work by Tinganelli and colleagues of LET-mediated kill painting (41), Sokol et al. extended the methodology to incorporate multiple ions and voxel-by-voxel target oxygenation data. Utilizing this plan with helium and oxygen ion beams, mean brainstem dose was reduced by 3–5% for helium and 10–12% for oxygen, respectively, with full biological optimization. Dosimetric validation of these particle species (76), validation of Monte Carlo modeling FLUKA code (77), and experimental validation of the resultant treatment planning tool against dosimetric measurements in water, have similarly been performed (78).

Kopp and colleagues followed with the “PaRticle thErapy using single and Combined Ion optimization StratEgies” (PRECISE) treatment planning system, allowing for delivery of single field multi-ion particle therapy treatments (40). They validated these plans across three patient cases, as well as in a murine glioma cell line, generating a highly uniform physical dose while reducing high dose averaged LET gradients in comparison with CIRT monotherapy. They found that biophysical stability in the target volume was similar to protons, while normal tissue dose was similar or improved *versus* helium dose planning, with < 1% deviation from the planned target RBE value.

## Challenges, Future Directions, and Conclusions

Significant considerations are required for the possible translation of these initial developments within MIRT to clinical treatment. Incorporation of lighter ions to reduce damage to target-adjacent normal tissues may risk underdosing relative single-beam irradiation. Careful multi-angle dose simulation, modeling, and validation is required. Center treatment throughput will require consideration: the NIRS has developed a system capable of source switching in under one minute, with ongoing development to reduce source switching to < 5 s. Treatment times could thereby only be limited by patient repositioning. Further details regarding a multi-ion clinical treatment system at the NIRS-QST are forthcoming.

Notably, dose-averaged LET is a macroscopic quantity, and may poorly approximate the physical reactions occurring at a cellular level (79). LET and RBE do not form a linear relationship, with track structure and microdosimetry potentially allowing an outsized increase in RBE with increases in LET (80, 81). Consequently, the translation of physical dose to a uniform cell-killing biological effect is model-dependent. Biological effects in varying tissues unique to any given ion may be as-yet unknown, and translation of physical dose and LET-optimized distributions to kill effect will require significant development.

To date, both the MKM and LEM models assume a normal oxygen pressure. As the key treatment targets for MIRT are focused on radioresistant and hypoxic tumors, evaluation of tissue oxygen conditions and resultant biological effects will be needed (82). Moreover, variation in biophysical models between ions (38, 83) requires reconciliation, including means by which to evaluate clinical uncertainty during treatment planning. As current dose plan arrangements encounter difficulty in intermodal translation (38), careful planning with MIRT is required so as not to exacerbate these issues during initial clinical trials. Further consideration of what range of LET provides the ideal clinical effect is also needed (84). Similarly, biological differences inherent to dose rate are currently being explored (i.e., FLASH); current biophysical modeling assumes a normal dose rate, and variations in treatment effect between dose rates may also influence future CPT and MIRT treatment. Developments within these areas of study will deserve careful note.

An external, international assessment of CIRT at NIRS was conducted in 2015, noting the significant promise of CIRT and recommending key consideration for methodologies improving patient throughput while reducing cost (7). These thoughts similarly inform efforts to translate MIRT from bench to bedside. Concerns regarding secondary cancer development with ion therapy remain, though a propensity score-weighted analysis comparing CIRT, conventional radiotherapy, and surgery for localized prostate cancer found a lower risk of subsequent primary cancer following CIRT *vs.* photon irradiation (20). Further verification will be needed as novel ion therapies are employed in treatment.

Particle irradiation has been studied for 70 years. Today, as the United States endeavors to construct its first heavy-ion capable facility and centers in Europe and Asia continue development of heavy-ion, multi-ion radiotherapy appears technically feasible for future treatment of radioresistant and hypoxic cancers. Robust international collaboration will be critical to produce dose modeling consensus, build upon the common borders of radiobiology and particle physics, and ensure access of the global population to novel treatments within radiation oncology. Significant technological and radiobiological progress has been made toward realizing initial trials for multi-ion radiotherapy, but more remains.

## Data Availability Statement

The original contributions presented in the study are included in the article/supplementary material. Further inquiries can be directed to the corresponding author.

## Author Contributions

DE: manuscript conception, writing, preparation TI: preparation. editing, review, figure preparation. DE, SF, SY and TS: conception, preparation, editing, review. All authors contributed to the article and approved the submitted version.

## Funding

Supported in part by Cancer Center Support (Core) Grant P30 CA016672 (PI, PWT Pisters) from the National Cancer Institute, National Institutes of Health, to The University of Texas MD Anderson Cancer Center.

## Conflict of Interest

The authors declare that the research was conducted in the absence of any commercial or financial relationships that could be construed as a potential conflict of interest.
